# Managing *Vibrio parahaemolyticus* and *Vibrio alginolyticus* Infections in the Whiteleg Shrimp (*Penaeus vannamei*): A Systematic Review

**DOI:** 10.3390/molecules30173620

**Published:** 2025-09-04

**Authors:** Nicolás Renato Jara-Medina, Ariana Cecibel Cedeño-Pinargote, Andrea Beltrán-Noboa, Eduardo Tejera, António Machado

**Affiliations:** 1 Laboratorio de Bacteriología, Instituto de Microbiología, Colegio de Ciencias Biológicas y Ambientales COCIBA, Universidad San Francisco de Quito USFQ, Calle Diego de Robles y Pampite, Quito 170901, Ecuador; nrjara@alumni.usfq.edu.ec (N.R.J.-M.); accedenop@alumni.usfq.edu.ec (A.C.C.-P.); 2 Grupo de Bioquimioinformática, Facultad de Ingeniería y Ciencias Agropecuarias Aplicadas, Universidad de Las Américas, Quito 170125, Ecuador; andreabeltran.n@outlook.com; 3 Escuela Superior Politécnica Agropecuaria de Manabí—Manuel Félix López, Carrera de Medicina Veterinaria, Calceta 130250, Manabí, Ecuador; 4 Centro de Biotecnologia dos Açores (CBA), Departamento de Biologia, Faculdade de Ciências e Tecnologia, Universidade dos Açores, 9500-321 Ponta Delgada, Portugal

**Keywords:** vibriosis, *Vibrio*-related infections, early mortality syndrome, antimicrobial agents, secondary metabolites, antibiofilm activity, systematic review

## Abstract

Background: This systematic review aims to evaluate the effectiveness of various treatments and strategies for managing infections caused by *Vibrio parahaemolyticus* and *Vibrio alginolyticus* in whiteleg shrimp (*Penaeus vannamei*). Shrimp aquaculture faces significant challenges from these pathogens, resulting in substantial economic losses. *Vibrio* species are known for their ability to form biofilms, enhancing their resistance to conventional treatments. Methods: The review follows the PRISMA guidelines, searching Scopus and PubMed databases for relevant studies on antibiotics and plant extracts used against these pathogens. Data were extracted and analysed to assess the effectiveness of different treatments, including antibiotics, plant extracts, and combined therapies. Results: The review found that while antibiotics remain widely used, the emergence of antibiotic-resistant strains necessitates alternative strategies. Plant extracts, rich in bioactive compounds such as flavonoids, showed promising antimicrobial activity. Combined therapies involving antibiotics and plant extracts were also explored for their potential to enhance treatment efficacy and reduce resistance. Conclusions: The findings underscore the importance of addressing biofilm formation in managing *Vibrio*-related infections and highlight the need for further research to develop sustainable and effective treatment protocols for shrimp aquaculture.

## 1. Introduction

Shrimp farming represents one of the most important aquaculture activities and has been established as an important socioeconomic promoter worldwide, especially in countries like Ecuador. Whiteleg shrimp (*Penaeus vannamei*) aquaculture constantly faces the challenge of diseases thanks to the conditions that promote the rapid growth of opportunistic bacteria such as many *Vibrio* species, with *Vibrio parahaemolyticus* and *Vibrio alginolyticus* being among the main pathogens associated with mortality and significant economic losses in shrimp and mollusk exporting countries [1,2]. These diseases caused by *V. parahaemolyticus* and *V. alginolyticus* species can result in losses ranging from 20% to 80% of the total production [3]. In addition, it is well known that *Vibrio* spp. can form biofilms. In this case, the species of interest demonstrate a high capacity to adhere to biotic and abiotic surfaces for biofilm formation, which makes them more resistant to conventional treatments and control [4].

*Vibrio* is a genus of motile, curved, Gram-negative bacteria found naturally in aquatic environments primarily forming multi-species biofilms [5]. *V. parahaemolyticus* is a halophilic marine bacterium, considered an emerging pathogen with a global distribution. It is the most prevalent shrimp pathogen in aquaculture, associated with recurrent outbreaks and substantial economic impact. *V. alginolyticus* is a moderately halophilic and mesophilic bacterium, being recognized as an epizootic pathogen responsible for severe outbreaks of diseases such as vibriosis in aquatic and marine animals [6,7]. Although some species of *Vibrio* are harmless or even beneficial to aquatic organisms, certain pathogenic strains can cause serious diseases in farmed shrimp. As previously mentioned, the culture disease that affects the greatest proportion is vibriosis, whose mortality can vary between 70 and 100%, and it damages the oral cavity and appendages of the shrimp [8]. Additionally, it is recorded that farmed shrimp also acquire other diseases due to the previously mentioned *Vibrio* species, such as acute hepatopancreatic necrosis disease (AHPND), zoea 2 syndrome, white feces disease (WFD), seagull syndrome (SGS), mysis mold syndrome, and bolitas syndrome, which are the most common reported in the literature [1,9,10,11,12]. Among these conditions, AHPND is probably the most impactful *Vibrio*-related infection in shrimp aquaculture, being responsible for severe economic losses worldwide, with estimates approaching USD 43 billion and up to 60% production losses in affected regions [13,14]. Clinically, AHPND (also known as early mortality syndrome) is characterized by reduced feeding, erratic swimming followed by lethargy, empty or pale/atrophic hepatopancreas, and rapid progression to high or even mass mortalities in cultured *P. vannamei* [15,16,17]. *Vibrio* species cause infection at all life stages (from eggs to brood stock). The adaptability of these bacteria to stress conditions, their rapid reproduction, their ability to generate resistance to antibiotics, and their ability to form biofilms pose a significant challenge for the aquaculture industry [18,19].

The presence of bacterial diseases caused by *V. parahaemolyticus* and *V. alginolyticus* represents a constant threat to the sustainability and profitability of whiteleg shrimp aquaculture [20]. These losses include direct mortality of infected shrimp, decreased growth and meat quality, as well as reduced feed conversion ratio, without considering the economic impact of these losses. Historically, Ecuador has had different massive outbreaks of systemic vibriosis that have harmed the economy and the production of *P. vannamei* shrimp, with mortality rates of 5 to 90%. Consequently, the economic problems in exports were severe and the Ecuadorian economy suffered a contraction, which led to an acute economic crisis within the shrimp industry worldwide [10,21]. Therefore, to control *Vibrio* infections in shrimp farms worldwide, various treatments and strategies have been implemented. Among the most common methods are the use of antibiotics, plant extracts, probiotics, disinfectants, immunotherapy, and improvements in management practices and biosecurity in production [22]. However, *V. parahaemolyticus* and *V. alginolyticus* can form biofilms and generate resistance to these treatments, which leads to persistence in aquaculture systems. These biofilms are microbial communities attached to solid surfaces, such as farm structures and the shrimp themselves [23]. Biofilms protect *Vibrio* species from disinfectants, shrimp immune systems, and other control factors, making their eradication and efficient control difficult to achieve nowadays [24,25,26].

This systematic review aims to collect and analyse relevant scientific studies that evaluate the effectiveness of the different treatments and strategies used to control diseases caused by *Vibrio* species in whiteleg shrimp farms. Additionally, the effectiveness of measures aimed at destabilizing and eliminating biofilms formed by these bacteria was examined, considering their influence on the persistence and resistance of *Vibrio* species in aquaculture systems.

This systematic review aims to synthesize current knowledge on the effectiveness of treatments used to control diseases caused by *Vibrio* spp. in whiteleg shrimp aquaculture. Particular attention is given to the role of biofilm formation, which represents a major challenge for therapeutic success. The overall objective is to identify which strategies, including antibiotics, plant-derived compounds, and combined approaches, are most effective, while also clarifying their limitations and outlining promising alternatives for sustainable disease management.

## 2. Results

### 2.1. Study Inclusion Criteria and Characteristics of the Eligible Studies

**Figure 1:**
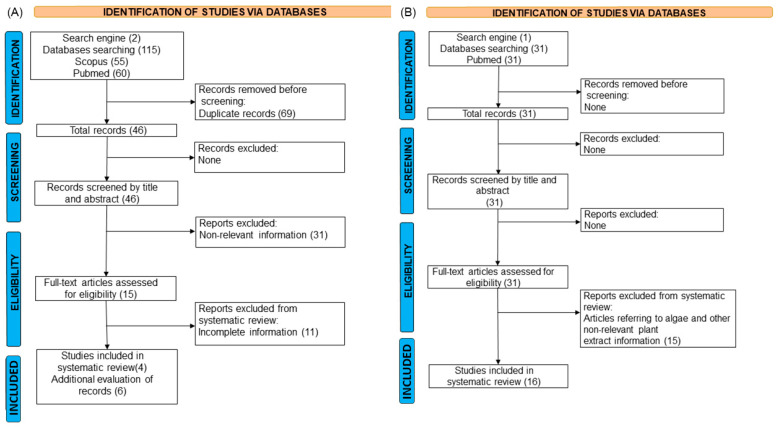
Prisma flow chart of the selection process for *Vibrio* treatments with antibiotics (**A**) and plant extracts (**B**).

A total of 46 studies with antibiotics were retrieved and, after the screening and eligibility steps, 4 full-length articles were selected, but the additional evaluation of records (references) of the initial set of 4 studies allowed us to select 6 studies more evaluating antibiotics against *Vibrio* species (see Figure 1A). Moreover, these additional studies also reported data of other *Vibrio* species besides the species of interest (i.e., *V. parahaemolyticus* and *V. alginolyticus*). These studies were included to provide complementary insights, given the limited availability of information on the target species. Their inclusion was justified by their use of comparable methodologies and their relevance to understanding broader *Vibrio*-related dynamics. On the other hand, a total of 31 studies with plant extracts were collected and 16 full-length articles were used, where no additional records were found. These studies met the inclusion criteria for the systematic review (see Figure 1B). The final dataset included studies from different global regions. All available and relevant data from each study were extracted, specifically focusing on *Vibrio* species, antibiotics, and plant extracts, applied concentrations, methodologies, and quantitative parameters (such as replicates, survival rate, mean, and standard deviation).

After applying the screening, eligibility, and quality assessment criteria, 26 studies addressing antibiotics and plant extracts were included in this systematic review (Figure 1).

### 2.2. General Effects of Vibrio Species in Penaeus vannamei

The general data of the selected antibiotic studies are shown in Table 1, which comprises studies conducted between 2012 and 2020 in several countries around the world, primarily in Asia (1/4) and South America (3/4). Furthermore, after checking additional records in the initial four studies, the other six studies were found between 2019 and 2023, also from Asia (5/6) and South America (1/6). Additionally, the disk diffusion assay was used to evaluate the effectiveness of antibiotics against *Vibrio* isolates. The general data of the selected plant extract studies are shown in Table 2, which comprises studies conducted between 2007 and 2021 in several countries around the world, mainly in Asia (15/16) and North America (1/16). *Vibrio* survival assays were used to evaluate the effectiveness of plant extracts against *Vibrio*. The tested strains of different *Vibrio* species were isolated from shrimp farming areas in different countries.

**Table 1:** General antibiotic information extracted from the dataset selected for the present systematic review.

Study	Region	Country	*Vibrio* Species	Antibiotics	Families	Susceptibility
[27,28]	South America	Brazil	*V. parahaemolyticus*	Oxytetracycline	Tetracyclines	Intermediate
			*V. parahaemolyticus*	Ampicillin, Colistin, Streptomycin	Penicillins, Aminoglycosides	Resistant
			*V. parahaemolyticus*	Penicillin, Tetracycline	Penicillins, Tetracyclines	Resistant
			Other *Vibrio* species: *V. brasiliensis*, *V. cholerae*, *V. coralliilyticus*, *V. navarrensis*, *V. vulnificus*, *V. xuii*, and *V. cholerae* ATCC 19582	Ampicillin, Aztreonam, Ceftriaxone, Cephalothin, Chloramphenicol, Ciprofloxacin, Gentamicin, Imipenem, Nalidixic acid, Nitrofurantoin, Penicillin, Streptomycin, Sulfamethoxazole-trimethoprim, Tetracycline	Penicillins, Cephalosporins, Aminoglycosides, Fluoroquinolones, Tetracyclines, Sulfonamides, Carbapenems, Monobactams, Amphenicols, Nitrofuranos	Sensitive
[23]	South America	Ecuador	*V. alginolyticus*	Nalidixic acid, Chloramphenicol, Ciprofloxacin, Enrofloxacin, Florfenicol, Fosfomycin, Furazolidone, Norfloxacin	Quinolone, Amphenicols, Fluoroquinolones Phosphonic acids, Nitrofurans	Sensitive
[1]	Asia	India	*V. parahaemolyticus*	Chloramphenicol, Gentamicin, Meropenem, Tetracycline, Trimethoprim/sulfamethoxazole	Amphenicols, Aminoglycosides, Carbapenems, Tetracyclines, Sulfonamide/Trimethoprim	Sensitive
**Other Studies**						
[29]	South America	Brazil	Other *Vibrio* species: *V. vulnificus*	Chloramphenicol, Ciprofloxacin, Norfloxacin	Amphenicols, Fluoroquinolones	Intermediate
			Other *Vibrio* species: *V. vulnificus*	Amikacin, Ampicillin, Enrofloxacin, Gentamicin, Neomycin, Penicillin, Tetracycline	Aminoglycosides, Penicillins, Fluoroquinolones, Tetracyclines	Resistant
[30]	Asia	Malaysia	*V. cholerae*	Streptomycin, Erythromycin, Tetracycline	Aminoglycosides, Macrolides, Tetracyclines	Sensitive
			*V. parahaemolyticus*	Bacitracin	Polypeptides	Intermediate
			*V. parahaemolyticus* Other *Vibrio* species: *V. cholerae*	Penicillins, Glycopeptides	Penicillins, Glycopeptides	Resistant
[5]	Asia	Korea	*V. parahaemolyticus*	Vancomycin, Amikacin, Cefepime, Cefotaxime, Ceftriaxone, Erythromycin, Imipenem, Streptomycin, Ticarcillin-clavulanic acid	Glycopeptides, Aminoglycosides, Cephalosporins, Macrolides, Carbapenems, Penicillin/Beta-lactamase	Sensitive
			*V. parahaemolyticus*	Amikacin, Ampicillin, Cefepime, Ceftazidime, Chloramphenicol, Polymyxin B, Tazobactam-piperacillin	Glycopeptides, Aminoglycosides, Cephalosporins, Macrolides, Penicillin/Beta-lactamase	Intermediate
			*V. parahaemolyticus*	Amikacin, Ampicillin, Cefepime, Ciprofloxacin, Clarithromycin, Doxycycline, Gentamicin, Kanamycin, Levofloxacin, Minocycline, Nalidixic acid, Penicillin, Sulfamethoxazole- trimethoprim, Tetracycline, Tobramycin	Aminoglycosides, Penicillins, Cephalosporins, Fluoroquinolones, Macrolides, Tetracyclines, Sulfonamides/Trimethoprim	Resistant
[31]	Asia	Singapore	*V. parahaemolyticus*	Ampicillin/sulbactam, Cefotaxime, Chloramphenicol, Ciprofloxacin, Sulfamethoxazole- trimethoprim, Tetracycline	Penicillin/Beta-lactamase, Cephalosporins, Chloramphenicols, Fluoroquinolones, Sulfonamides/Trimethoprim, Tetracyclines	Sensitive
			*V. parahaemolyticus*	Ampicillin, Penicillin	Penicillins	Resistant
[32]	Asia	Vietnam	*V. parahaemolyticus*	Chloramphenicol, Gentamicin, Kanamycin, Nalidixic acid, Oxacillin, Tebipenem	Amphenicols, Aminoglycosides, Quinolones, Penicillins, Carbapenems	Sensitive
[33]	Asia	China	*V. alginolyticus*	Chloramphenicol, Doxycycline, Minocycline	Chloramphenicols, Tetracyclines	Sensitive
			*V. alginolyticus*	Amikacin, Cefperazone, Gentamicin, Kanamycin, Tetracycline	Aminoglycosides, Cephalosporins, Tetracyclines	Intermediate
			*V. alginolyticus*	Ampicillin, Carbenicillin, Cefazolin, Cefperazone, Ceftazidime, Ceftriaxone, Cefuroxime, Cephalexin, Cephradine, Ciprofloxacin, Clarithromycin, Erythromycin, Furazolidone, Nalidixic acid, Ofloxacin, Oxacillin, Penicillin, Piperacillin, Polymyxin B, SMZ/TMP, Vancomycin	Penicillins, Cephalosporins, Fluoroquinolones, Macrolides, Nitrofurans, Quinolones, Polymyxins, Sulfonamides/Trimethoprim, Glycopeptides	Resistant

All tests were carried out using the disk diffusion method.

**Table 2:** General plant extract information extracted from the dataset of the present systematic review.

Study	Region	Country	*Vibrio* Species	Plant	Effectiveness *	Type of Extract
[34]	North America	Mexico	*V. parahaemolyticus*, *V. alginolyticus*	*Caulerpa sertularioides*	High	Methanolic extract
[35]	Asia	Indonesia	*V. parahaemolyticus*	*Eleutherine bulbosa*	Intermediate/High	Ethanolic extract
[36]	Asia	Korea	*V. alginolyticus*	*Rubus coreanus*	High	Ethanolic extract
[37,38]	Asia	Malaysia	*V. parahaemolyticus*	*Pandanus tectorius*	Intermediate/High	Methanolic extract
[39]	Asia	Taiwan	*V. alginolyticus*	*Cinnamomum kanehirae*	Intermediate/High	Water extract
[40]	Asia	Taiwan	*V. alginolyticus*	*Gelidium amansii*	High	Water extract
[41]	Asia	Taiwan	*V. alginolyticus*	*Gynura bicolor*	Intermediate/High	Water extract
[42]	Asia	Taiwan	*V. alginolyticus*	*Gynura bicolor*	Intermediate	Water extract
[43]	Asia	Taiwan	*V. alginolyticus*	*Phyllanthus amarus*	Intermediate/High	Water extract
[44]	Asia	Taiwan	*V. parahaemolyticus*	*Psidium guajava*	Intermediate/High	Water extract
[45,46]	Asia	Taiwan	*V. parahaemolyticus* and *V. alginolyticus*	*Moringa oleifera*	Intermediate/High	Water extract
[47]	Asia	Taiwan	*V. alginolyticus*	*Theobroma cacao*	High	Extract from fresh peels
[48]	Asia	Thailand	*V. parahaemolyticus*	*Macleaya cordata*	High	Water extract
[49]	Asia	Vietnam	*V. parahaemolyticus* Other *Vibrio* species: *V. harveyi*	*Rhodomyrtus tomentosa*	Intermediate	Seed extract

* Effectiveness (survival rate of shrimps): Intermediate (Between 50 and 70%), Intermediate/High (Between 70 and 80%), and High (Up to 90%).

In this systematic review, four studies focused on *V. parahaemolyticus* and *V. alginolyticus*, while six additional studies involving other *Vibrio* species were included to complement information on antibiotic use within the genus. Table 1 summarizes the 55 antibiotics reported across the 10 studies, which were grouped into 16 antibiotic families and tested against 11 *Vibrio* species. The primary species analyzed were *V. alginolyticus* and *V. parahaemolyticus*, although other species such as *V. brasiliensis*, *V. cholerae*, *V. coralliilyticus*, *V. neptunius*, *V. vulnificus*, and *V. xuii* were also evaluated, providing broader context without diminishing the relevance of the two focal species. Regarding susceptibility, *V. alginolyticus* generally exhibited greater sensitivity to antibiotics, whereas *V. parahaemolyticus* showed comparatively higher resistance.

Plant extracts were also included in this systematic review, with 16 studies selected for analysis. Table 2 summarizes the 13 plant extracts tested against *V. parahaemolyticus*, *V. alginolyticus*, and, in one case, *V. harveyi*. Survival ranges of shrimp infected with these *Vibrio* species were evaluated to assess the in vivo efficacy of the extracts. Overall, the plant extracts demonstrated good protective effects, with most water-, methanol-, and ethanol-based preparations yielding intermediate to high shrimp survival (70–90%). Among the tested extracts, only the seed extract produced an intermediate shrimp survival rate (up to 50%). The dataset encompassed studies from diverse global regions, and relevant information was systematically extracted, including *Vibrio* species, treatments (antibiotics and plant extracts), applied concentrations, methodologies, and quantitative parameters such as replicates, survival rate, mean, and standard deviation.

### 2.3. Prevalence of Vibrio-Related Infections in P. vannamei and Their Geographical Distribution

Pacific white shrimp (*P. vannamei*), also known as whiteleg shrimp, is one of the most farmed and traded shrimp species worldwide. The contamination of this type of shrimp with *V. parahaemolyticus* and *V. alginolyticus* as well as other *Vibrio* species has been a problem for a long-time and currently has serious repercussions on the industry. It is well-known that *Vibrio* species can form mature and mostly resistant biofilms [22,50]. Table 3 shows how the prevalence of *Vibrio* is distributed in the shrimp samples at a geographic level. In our database previously explained in Table 1 and Table 2, of the 10 and 16 articles on the use of antibiotics and plant extracts against *Vibrio* strains in this review, respectively, all samples originated from shrimp farming areas with the presence of different species of *Vibrio*, with *V. parahaemolyticus* and *V. alginolyticus* being the ones that occurred in the greatest proportion, and regarding infections in the *P. vannamei* shrimp, it was observed that for the most part, the most common condition present was vibriosis. Studies in Asia and South America showed infection rates relatively close in numbers. However, the four studies in South America (Brazil and Ecuador) revealed a lower number of *Vibrio* strains for antibiotic efficacy evaluation [23,27,28,29].

**Figure 2:**
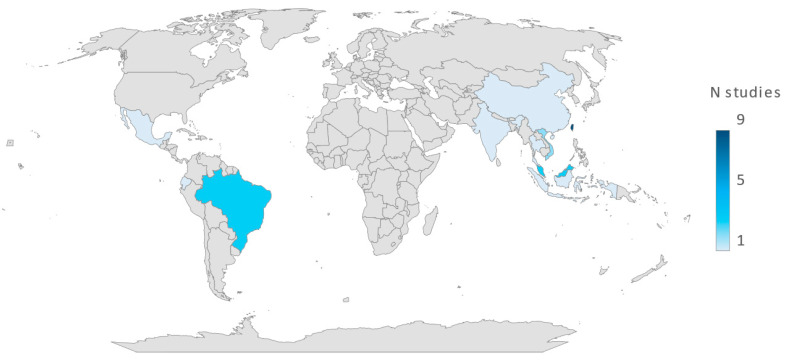
Summary of global studies reporting the prevalence of *Vibrio* species and related infections.

The prevalence of *Vibrio* types in shrimp varied significantly among studies from different regions and countries, especially between South America and North America. On the other hand, Asia reported an intermediate prevalence of *Vibrio* presence of around 74%. However, the limited number of studies may lead to biased conclusions, underscoring the need for additional data. Figure 2 shows that the dataset included few countries and most of them merely reported one study, except for Taiwan (9 studies), Malaysia (3 studies), Brazil (3 studies), Vietnam (2 studies), and Korea (2 studies).

**Figure 3:**
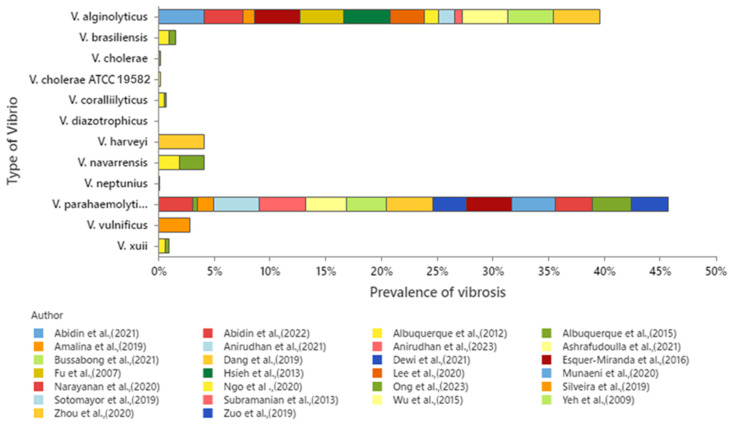
Percentage of *Vibrio* types present in the dataset. *Vibrio cholerae* ATCC 19582 is included as a reference strain used for comparison in antimicrobial susceptibility testing [1,5,23,27,28,29,30,31,32,33,34,35,36,37,38,39,40,41,42,43,44,45,47,48,49,51].

Although it is established that the review focuses on *V. parahaemolyticus* and *V. alginolyticus*, the analysis of the database indicates that a total set of 12 *Vibrio* species is presented, of which 11 *Vibrio* species were used for studies of antibiotics, while only 3 *Vibrio* species were used in plant extract studies. The prevalence of *Vibrio* types for each *Vibrio* species in the dataset (26 articles) was mainly attributed to *V. parahaemolyticus* (46%), followed by *V. alginolyticus* (43%), and a minority of 11% to the remaining species (Figure 3).

The data collected showed that numerous *Vibrio* species exhibited resistance to different antibiotics, whereas most plant extracts demonstrated consistent antimicrobial activity. Although all samples were obtained from isolates associated with shrimp culture, one study employed *V. cholerae* ATCC 19582 as a reference strain [28], while other isolates originated from previously published reports [37,45]. This strain is a standard laboratory reference used in antimicrobial susceptibility testing and does not represent a distinct subspecies. Reported prevalence of *Vibrio* types, particularly *V. parahaemolyticus* and *V. alginolyticus*, varied considerably among studies, underscoring the need for broader and more standardized investigations to establish their true global prevalence.

### 2.4. Antibiotics Studies

In current aquaculture practices, the most common strategy to combat *Vibrio* infections is the use of broad-spectrum antibiotics. According to the literature, the antibiotics most frequently applied in shrimp farming include cephalothin (5.8%), ampicillin (9.9%), penicillin (8.9%), ceftriaxone (6.3%), aztreonam (5.8%), and tetracycline (9.4%). In our dataset of 10 articles, a wide variety of antibiotics were identified across different studies. To facilitate interpretation, the analysis was grouped by antibiotic families, since members of the same family share similar mechanisms of action against *Vibrio*. Table 4 provides an overview of the conditions, concentrations, and susceptibility profiles reported among *Vibrio* species. For example, 14 antibiotic families were tested against *V. alginolyticus*, using varying concentrations under culture temperatures between 28 and 35 °C for 24 h. This species showed resistance in 8 families, sensitivity in 5 families, and an intermediate response in 1 family. *V. parahaemolyticus* was tested against 13 antibiotic families under similar conditions (35–37 °C for 24 h) and exhibited resistance, intermediate responses, and sensitivity across all families. Notably, the proportion of sensitive strains in *V. parahaemolyticus* was higher, with 47.1% of isolates responding to most antibiotic families.

**Table 4:** Antibiotic treatments and conditions against *Vibrio* species.

*Vibrio* Species	Antibiotics Families	Concentration (μg)	Conditions	N° Strains Susceptibility	Studies
*V. alginolyticus*	Aminoglycosides	30	28 °C-24 h	3 Intermediate	[23,27,33]
	Carbapenems	30	28 °C-24 h	1 Resistant	
	Cephalosporins	30–75	28/35 °C-24 h	3 Sensitive 1 Intermediate 6 Resistant	
	Fluoroquinolones	5–10	30 °C-24 h 28 °C-24 h	4 Sensitive 3 Resistant	
	Glycopeptides	30	28 °C-24 h	1 Resistant	
	Macrolides	15	28 °C-24 h	2 Resistant	
	Monobactam	30	35 °C-24 h	1 Sensitive	
	Nitrofuran	100	30 °C-24 h	1 Sensitive 1 Resistant	
	Penicillins	1–100 10 U	28/35 °C-24 h	2 Sensitive 6 Resistant	
	Peptide	200 (l U)	28 °C-24 h	1 Resistant	
	Phenicole	30	28/30 °C-24 h	3 Sensitive	
	Phosphonate	10	30 °C-24 h	1 Sensitive	
	Sulfonamide	23.75/1.25	28 °C-24 h	1 Resistant	
	Tetracyclines	30	28/35 °C-24 h	4 Sensitive 1 Intermediate 2 Resistant	
*V. parahaemolyticus*	Aminoglycosides	10–30	37 °C-24 h	69 Sensitive 63 Intermediate 202 Resistant	[1,5,27,30,31,32]
	Beta-lactamase	110	37 °C-24 h	16 Sensitive 44 Intermediate 2 Resistant	
	Carbapenems	10–30	37 °C-24 h	73 Sensitive 16 Intermediate 1 Resistant	
	Cephalosporins	30	35 °C-24 h 37 °C-24 h	165 Sensitive 129 Intermediate 65 Resistant	
	Fluoroquinolones	5–30	37 °C-24 h	19 Sensitive 53 Intermediate 144 Resistant	
	Glycopeptides	30	37 °C-24 h	62 Resistant	
	Macrolides	2–15	37 °C-24 h	40 Sensitive 22 Intermediate 62 Resistant	
	Monobactam	30	35 °C-24 h	1 Sensitive	
	Penicillins	5–20 (10 U)	35/37 °C-24 h	62 Sensitive 42 Intermediate 158 Resistant	
	Peptide	10–300	37 °C-24 h	8 Sensitive 50 Intermediate 18 Resistant	
	Phenicole	30	37 °C-24 h	59 Sensitive 30 Intermediate 2 Resistant	
	Sulfonamide	1.24–25	37 °C-24 h	28 Sensitive 26 Intermediate 36 Resistant	
	Tetracyclines	30	35/37 °C-24 h	32 Sensitive 9 Intermediate 174 Resistant	
**Other Types of *Vibrio* Analyzed**					
*V. cholerae*	Aminoglycosides	10	37 °C-24 h	11 Sensitive 8 Intermediate 4 Resistant	[27,28,30]
	Cephalosporins	30	35 °C-24 h	28 Sensitive 1 Intermediate	
	Glycopeptides	30	37 °C-24 h	8 Sensitive 3 Intermediate 2 Resistant	
	Macrolides	15	37 °C-24 h	2 Sensitive 1 Intermediate 10 Resistant	
	Monobactam	30	35/37 °C-24 h	22 Sensitive 6 Intermediate	
	Penicillins	10 (10 U)	35/37 °C-24 h	28 Sensitive 7 Intermediate 8 Resistant	
	Thetracyclines	30	35/37 °C-24 h	1 Sensitive 2 Intermediate 11 Resistant	
*V. cholerae* ATCC 19582	Aminoglycosides	10	NA	26 Sensitive	[28]
	Carbapenems	10	NA	26 Sensitive	
	Cephalosporins	30	NA	10 Sensitive 3 Intermediate 3 Resistant	
	Fluoroquinolones	5 30	NA	25 Sensitive 1 Intermediate	
	Nitrofurans	300	NA	26 Sensitive	
	Penicillins	10	NA	17 Sensitive 9 Resistant	
	Phenicole	30	NA	26 Sensitive	
	Sulfonamide	25	NA	26 Sensitive	
	Tetracyclines	30	NA	26 Sensitive	
*V. brasiliensis*	Cephalosporins	30	35 °C-24 h	2 Sensitive 1 Intermediate	[27]
	Monobactam	30	35 °C-24 h	1 Sensitive	
	Penicillins	10 (10 U)	35 °C-24 h	1 Sensitive 1 Intermediate 1 Resistant	
	Tetracyclines	30	35 °C-24 h	1 Sensitive	
*V. coralliilyticus*	Cephalosporins	30	35 °C-24 h	2 Sensitive	[27]
	Monobactam	30	35 °C-24 h	1 Sensitive	
	Penicillins	10 (10 U)	35 °C-24 h	1 Sensitive 1 Resistant	
	Tetracyclines	30	35 °C-24 h	1 Sensitive	
*V. diazotrophicus*	Cephalosporins	30	35 °C-24 h	2 Sensitive	[27]
	Monobactam	30	35 °C-24 h	1 Sensitive	
	Penicillins	10 (10 U)	35 °C-24 h	2 Sensitive	
	Tetracyclines	30	35 °C-24 h	1 Sensitive	
*V. navarrensis*	Cephalosporins	30	35 °C-24 h	2 Sensitive 2 Intermediate	[27]
	Monobactam	30	35 °C-24 h	1 Sensitive 1 Intermediate	
	Penicillins	10 (10 U)	35 °C-24 h	1 Sensitive 1 Intermediate 1 Resistant	
	Tetracyclines	30	35 °C-24 h	1 Sensitive	
*V. vulnificus*	Aminoglycosides	10–30	37 °C–24 h	2 Sensitive 2 Intermediate 1 Resistant	[27,29]
	Cephalosporins	30	35 °C-24 h	3 Sensitive	
	Fluoroquinolones	5	37 °C–24 h	3 Resistant	
	Monobactam	30	35 °C-24 h	2 Sensitive	
	Penicillins	10 (10 U)	35 °C-24 h 37 °C–24 h	4 Sensitive 1 Intermediate 2 Resistant	
	Phenicole	30	37 °C–24 h	1 Resistant	
	Tetracyclines	30	35 °C-24 h 37 °C–24 h	1 Sensitive 2 Resistant	
*V. neptunius*	Cephalosporins	30	35 °C-24 h	2 Sensitive	[27]
	Monobactam	30	35 °C-24 h	1 Sensitive	
	Penicillins	10 (10 U)	35 °C-24 h	2 Sensitive	
	Tetracyclines	30	35 °C-24 h	1 Sensitive	
*V. xuii*	Cephalosporins	30	35 °C-24 h	2 Sensitive 1 Intermediate	[27]
	Monobactam	30	35 °C-24 h	1 Sensitive	
	Penicillins	10 (10 U)	35 °C-24 h	1 Sensitive 1 Intermediate 1 Resistant	
	Tetracyclines	30	35 °C-24 h	1 Sensitive	

**Figure 4:**
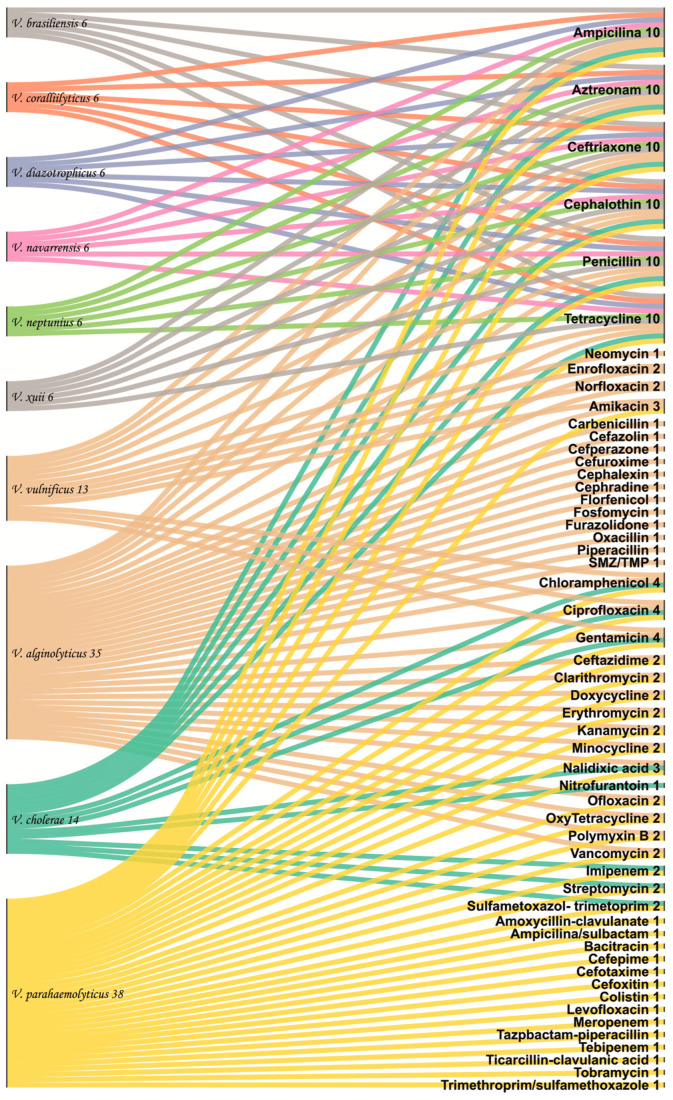
Interaction of antibiotic use in *Vibrio* species on the total dataset of the present study.

Figure 4 shows the frequency of antibiotic families used against *Vibrio* species. The interactions of antibiotics used against *Vibrio* species demonstrate a greater number of antibiotics used against *V. parahaemolyticus*, *V. alginolyticus*, and *V. cholerae*. More accurately, 10 antibiotic families were found to be more effective against *V. alginolyticus*. However, only 3 antibiotic families (carbapenems, cephalosporins, and phenicols) were more efficacious against *V. parahaemolyticus*. Furthermore, phenicol, monobactam, and cephalosporins families demonstrated treatment effectiveness against both *Vibrio* species. In contrast, eight antibiotic families proved to be ineffective against *Vibrio* species, specifically peptide, aminoglycosides, fluoroquinolones, glycopeptides, macrolides, penicillins, sulphonamides, and tetracyclines.

### 2.5. Plant Extract Evaluation

Dietary supplementation with herbs, oils, and plant extracts is a good strategy to combat different bacteria in the food industry nowadays. This strategy is one of the techniques that has the greatest use and research since it can improve the resistance of shrimps to infections of different types of *Vibrio* (such as vibriosis), the healing status, and growth performance. Plants are rich in a wide variety of secondary metabolites and bioactive compounds such as flavonoids, phenolic acid, alkaloids, tannins, and terpenoids [51,52]. Supplementation with plant extracts in shrimp diet improves their health status, and it is well known that herbal medicines can regulate gut microbial composition and its secretion, as well as activate the immune system of shrimp.

#### 2.5.1. Plant-Related Compounds Analysis

In the initial literature search, 31 articles were found evaluating the effects of plant-related compounds on pathogens, of which 16 full articles were analyzed. The compounds belonged to numerous plants, more exactly *Psidium guajava*, *Pandanus tectorius*, *Phyllanthus amarus*, *Moringa oleifera*, *Eleutherine bulbosa*, *Scutellaria baicalensis*, *Salvinia cucullata*, *Macleaya cordata*, *Gynura bicolor*, *Theobroma cacao*, *Rhodomyrtus tomentosa*, *Rubus coreanus*, and *Cinnamomum kanehirae*.

**Figure 5:**
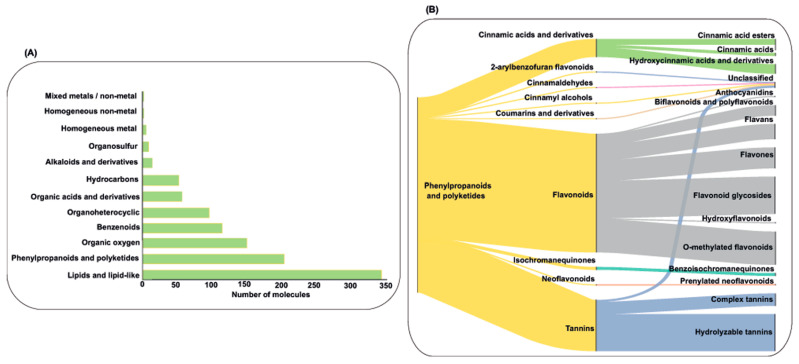
Illustration of the number of compounds in each chemical class (**A**) and representative diagram of the abundance of molecule subclasses into the phenylpropanoids and polyketides chemical class (**B**).

After consulting five databases for chemical compounds present in these plants, no information was found for *S. cucullata* and *C. kanehirae*. Across the remaining 11 plants, however, a total of 1082 unique molecules were identified. Among these, 11 were inorganic compounds, while 1071 were organic. The distribution of organic compounds was as follows: *E. bulbosa* (*n* = 58), *G. bicolor* (*n* = 41), *M. cordata* (*n* = 24), *M. oleifera* (*n* = 289), *P. tectorius* (*n* = 63), *P. amarus* (*n* = 154), *P. guajava* (*n* = 688), *R. tomentosa* (*n* = 55), *R. coreanus* (*n* = 61), *S. baicalensis* (*n* = 240), and *T. cacao* (*n* = 186). Figure 5 illustrates that most of the identified compounds belong to the phenylpropanoids and polyketides, along with the lipids and lipid-like classes. The abundance of lipids reflects the emphasis of many plant chemical characterizations on essential oils. The phenylpropanoids and polyketides include diverse flavonoids, which are of particular interest due to their antimicrobial properties.

**Figure 6:**
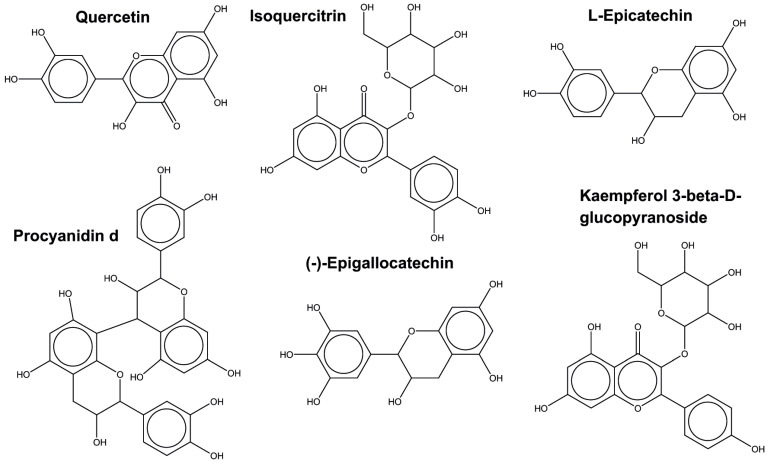
Structural representation of the most common flavonoids found across the plants under our study set.

In the flavonoid subclass, 210 unique compounds are distributed across 8 of the 11 plants in which chemical compounds were identified, more exactly *E. bulbosa*, *M. oleifera*, *P. tectorius*, *P. amarus*, *P. guajava*, *R. tomentosa*, *R. coreanus*, *S. baicalensis*, and *T. cacao*. Among these 210 compounds, the most commonly found molecules across the plants are presented in Figure 6.

Moreover, quercetin and isoquercitrin are regularly found in 6 and 5 plants, respectively, being structurally similar to kaempferol and its derivatives. A similar chemical scaffold is also found between epicatechin and epigallocatechin, while procyanidin (from the polyflavonoids subclass) is the most structurally different. Interestingly, all these molecules have a well-known antimicrobial effect.

#### 2.5.2. Antibacterial Activity by Plant Extracts

In our dataset (16 studies; Figure 1B), most extracts were aqueous (10/16), followed by methanol (3/16), ethanol (2/16), and a single seed extract (1/16). These preparations were obtained from different plant species, each contributing distinct properties and bioactive compounds. Table 5 summarizes the range of plants tested at varying concentrations and their antibacterial activity against the *Vibrio* species of interest.

**Table 5:** Efficiency of plant extract treatments on shrimp infected with different *Vibrio* species.

*Vibrio* Species	Plant Extracts	Concentration	Conditions	N° Strains Susceptibility	Studies
*V. alginolyticus*	*Caulerpa sertularioides*	300 mg/L	25 ± 5 °C-7 days	1 sensitive	[34,36,39,40,41,42,43,45,47,51]
	*Cinnamomum kanehirae*	2 μg/shrimp	28 °C-4 days	1 intermediate 1 sensitive	
	*Gelidium amansii.*	0.5–2 g/kg	28 ± 1 °C-6 days	1 sensitive	
	*Gynura bicolor*	0.5–2 g/kg (diet) 2–8 μg/g	27 ± 1 °C-28 days 27 ± 1 °C-2 days	1 intermediate 1 sensitive	
	*Rubus coreanus*	0.25–0.5% RcEE diet	25 ± 0.5 °C-7 days	1 sensitive	
	*Theobroma cacao*	10–40 μg/shrimp	28 ± 1 °C-6 days	1 sensitive	
	*Moringa oleifera*	1.25–5 g/kg	35 °C-24 h	1 sensitive	
	*Phyllanthus amarus*	10–40 g/kg	27 ± 1 °C-56 days	3 sensitive	
*V. parahaemolyticus*	*Caulerpa sertularioides*	300 mg/L	25 ± 5 °C-7 days	1 sensitive	[34,35,37,38,44,45,48,51]
	*Eleutherine bulbosa*	1.25–25 g/kg	28 ± 1 °C-7 days	1 intermediate 1 sensitive	
	*Macleaya cordata*	100–300 mg/kg	26 ± 1 °C-30 days	sensitive	
	*Moringa oleifera*	1.25 g/kg 2.5 g/kg 5.0 g/kg	35 °C-24 h	sensitive	
	*Pandanus tectorius*	0.5–6 g/L	28 °C-24 h	1 sensitive	
	*Psidium guajava*	1–10 g/kg	27 ± 2 °C-7 days	sensitive	
	*Rhodomyrtus tomentosa*	3500 μg/disc	25 ± 1 °C-7 days	1 sensitive	
**Other Types of *Vibrio* Analyzed**					
*V. harveyi*	*Rhodomyrtus tomentosa*	3500 μg/disc	25 ± 1 °C-7 days	2 sensitive	[49]

RcEE diet–*Rubus coreanus* ethanolic extract.

**Figure 7:**
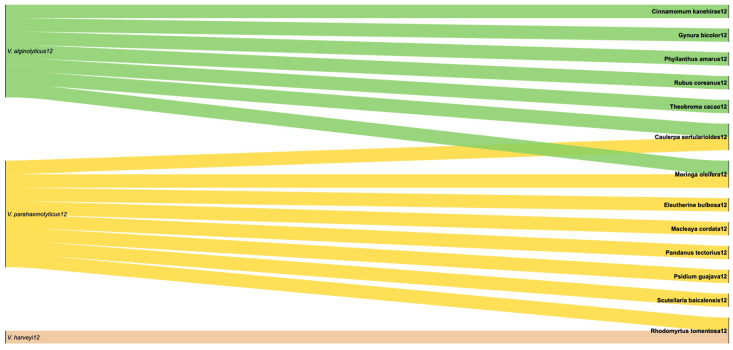
Representative illustration of the interactions of plant extracts used against *Vibrio* species in our study set.

For *V. alginolyticus*, eight different extracts were tested across varied concentration ranges and units, underscoring the lack of standardization in experimental protocols. Nevertheless, most studies were performed under comparable conditions (25–28 °C, 24 h up to 56 days) and consistently reported 1–3 sensitive strains per study, with occasional intermediate responses. For *V. parahaemolyticus*, seven extracts from different plants were evaluated under similar conditions (25–35 °C, 24 h up to 7 days). Here, sensitivity was again the dominant outcome, with only one intermediate response reported for *E. bulbosa* extract [35]. In addition, *R. tomentosa* extract was tested against *V. harveyi* at 25 °C for 7 days, yielding two sensitive strains. Figure 7 illustrates the frequency of each plant extract tested against the different *Vibrio* species. Notably, only two extracts, *C. sertularioides* [34] and *M. oleifera* [45,51], were evaluated against both *V. parahaemolyticus* and *V. alginolyticus*, while *R. tomentosa* was simultaneously tested against *V. parahaemolyticus* and *V. harveyi* [49]. This limited overlap in plant selection highlights a research gap; despite encouraging activity, reproducibility remains hindered by heterogeneous dosing and reporting. Future work should prioritize standardized protocols and cross-species testing to identify extracts with consistent and scalable efficacy in aquaculture.

### 2.6. Strategies for Vibrio-Related Infections in Penaeus vannamei

It is well established that each strategy for controlling *Vibrio*-related infections has its own advantages and drawbacks. When evaluating the activity of different approaches against *V. parahaemolyticus*, *V. alginolyticus*, and other Vibrio species affecting whiteleg shrimp (*P. vannamei*), it is essential to consider this diversity and its practical implications (see Table 5).

#### 2.6.1. Strengths and Limitations of Current Strategies

Several studies have analyzed the benefits and drawbacks of the main strategies applied to whiteleg shrimp (*P. vannamei*) for the management of *Vibrio*-related infections. Among the 26 articles evaluated in this review, the most commonly reported approaches include antibiotics, combined therapies, plant-based or natural treatments, and probiotics.

Table 6 summarizes the current strategies used to mitigate and prevent infections by *V. parahaemolyticus*, *V. alginolyticus*, and other *Vibrio* species in aquaculture, highlighting both their advantages and limitations. Depending on shrimp density and larval stage, different approaches may be selected. At present, antibiotics remain the most common method due to the availability of diverse compounds suitable for large tanks and early larval stages. Their main limitation, however, is the variation in effective concentrations depending on the antibiotic type and country. Moreover, different *Vibrio* strains can adapt to environmental conditions and harbor resistance genes, contributing to the widespread problem of resistance to multiple antibiotic families [53]. This challenge has driven the development of combined strategies, such as pairing effective antibiotics with plant extracts or incorporating probiotics with plant-derived compounds, to reduce resistance and improve protection. Although further studies are needed to fully characterize their antimicrobial benefits in shrimp, promising results have already been reported for controlling *Vibrio*-related infections in small tanks, larval stages, and adult shrimp [35,51].

**Table 6:** Evaluation of strengths and limitations of the main strategies to mitigate and prevent *Vibrio*-related infections in shrimp *P. vannamei*.

Strategies	Description	Strengths	Limitations	References
Antibiotics	Antibiotics are commonly used in aquaculture both for prophylactic purposes and to treat bacterial infections. The most used are tetracyclines, quinolones, phenicols, macrolides, and sulfonamides among others. They are usually administered directly in balanced food or water. Frequent and indiscriminate use is related to the development of bacterial resistance.	Establish adequate doses and administration times according to shrimp species and production stage. Rotate different families of antibiotics to avoid resistance. Use responsibly, only when necessary, based on laboratory diagnosis. Improve farming conditions to reduce stressful factors that promote infections.	Different concentrations of antibiotics in the aquaculture industry depending on the country-quality requirements and antibiotic residues in the final product The emergence of antibiotic-resistant *Vibrio* strains that persist in the aquaculture environment. Risk of transfer of resistance genes to human pathogenic bacteria through the food chain. Effects on other organisms in the aquatic ecosystem such as fish, mollusks, and plankton. Generation of antibiotic residues in shrimp tissue that reach the consumer.	[27]
Combined therapy (antibiotics plus plant extracts)	The most effective were furazolidone, ciprofloxacin, chloramphenicol, norfloxacin, nalidixic acid, florfenicol, and fosfomycin (inhibited the growth of most or all isolated strains). The effectiveness of essential oil EO1 (oregano oil extract, inhibited 100% of strains).	Furazolidone, ciprofloxacin, chloramphenicol, norfloxacin, nalidixic acid, florfenicol, and fosfomycin, enrofloxacin, inhibited most or all *Vibrio* strains. Oregano oil extract inhibited all *Vibrio* strains at low concentrations. Plant essential oils (EOs) combined with antibiotics: EOs can enhance the efficacy of antibiotics against multidrug-resistant pathogens.	Many antibiotics are not approved for use in aquaculture. Also, antimicrobial resistance was found in some strains. Some plant extracts exhibit toxicity, and their mechanisms of action are not well studied. Further, in vivo studies are required to confirm effectiveness. Effective in vitro doses may not correlate with the response in shrimp. More research is needed on specific antimicrobial action mechanisms and synergies with other compounds.	[23,53,54]
Plant extracts	Many medicinal plants have antibacterial activity against *Vibrio* species. Single extracts or a mixture of extracted compounds are used to treat vibriosis in shrimp. There are different types of plant extracts, among them the most used are aqueous, ethanolic, and methanolic.	According to the bibliography, the use of different types of plant extracts has the benefit of shrimp, improving growth and promoting immune activity. Additionally, it is a strategy that provides sustainable, ecological, and safe compounds, such as alkaloids, saponins, terpenoids, and flavonoids, to replace chemical compounds and antibiotics in aquaculture, being a solution for the antibiotic resistance that is currently observed in the industry.	The greatest limitation of this technique is the lack of more studies that apply it in vivo, to be able to generalize the results of efficacy and safety for the host (shrimp) because it has been reported that various plant extracts exhibit toxicity, so their mechanisms of action must be studied in greater depth. In addition, further research must be carried out to be able to make a protocol depending on the type of extract, and the larval stage of the shrimp.	[35,55]
Probiotics	This technique consists of administering probiotics, prebiotics, postbiotics, and synbiotics to the shrimp through the diet to combat or prevent *Vibrio* species.	The use of pro-, post-, pre-, and synbiotics in aquaculture has been considered effective food supplements that are prescribed to improve growth, antioxidant status, immunity, and resistance capacity to diseases such as vibriosis in different species of shrimp and other seafood.	The most significant disadvantage to using pro-, post-, pre-, and synbiotics is that they are often unable to maintain themselves and require supplementation regularly, which results in making this strategy less cost-effective.	[51,56,57]

#### 2.6.2. Comparative Cost and Efficiency

Beyond their biological activity, the choice of treatment strategies in aquaculture also depends on cost and efficiency, both of which determine their feasibility for large-scale application. A comparative analysis of these parameters highlights important differences among antibiotics, plant-derived treatments, probiotics, and emerging alternatives (see Table 7).

**Table 7:** Comparative cost and efficiency of main strategies to mitigate *Vibrio*-related infections in *P. vannamei*.

Strategy	Representative Agent(s)/Study	Dose/Usage (as Reported)	Reported Efficiency vs. *Vibrio*	Cost Category *	Notes/Caveats	References
Antibiotics	Oxolinic acid + oxytetracycline	50 mg OA/kg + 100 mg OTC/kg feed for 5 days; challenged with *V. parahaemolyticus*	Survival higher than control; combination therapy most effective	Medium–High	Effective in reducing mortality but associated with antimicrobial resistance (AMR), residue concerns, and monitoring costs	[58]
Combined therapy (antibiotics plus plant extracts)	Enrofloxacin (ENR) + San-Huang-San (SHS)	10–40 mg ENR/kg + 250–1000 mg SHS/kg feed for 5 days; challenged with AHPND-causing *V. parahaemolyticus*	Survival higher than with ENR or SHS alone; combination therapy significantly reduced mortality and improved immune response.	Medium.	Combination improves survival and immune response while allowing a reduction in antibiotic dose; still consider risks of AMR, standardization of herbal extract, dosing regimen, and short duration (5 days) → longer-term effects not assessed.	[59]
Plant extracts	Psidium guajava leaf extract	5 g/kg feed for 28–56 days; challenge with *V. parahaemolyticus*	Survival ~72% at optimal dose; improved growth and immune markers	Low–Medium	Promising alternative with immune-stimulating and eco-friendly properties; however, dose optimization and in vivo safety studies are needed	[44]
Probiotics	*Lactobacillus paracasei* or *Bifidobacterium longum*	Fed for 28 days; challenge with *V. parahaemolyticus*	Survival 67–73% depending on species	Low–Medium	Enhance immune responses and growth; generally cost-effective but require continuous supplementation	[60]

* Cost categories are qualitative, derived from peer-reviewed literature on input prices, application requirements, and biosecurity needs, not vendor pricing. Antibiotics are considered medium–high cost due to drug expenses, residue monitoring, and antimicrobial resistance (AMR) mitigation. Combined therapies (antibiotics + plant extracts) are classed as medium, since herbal supplements may reduce antibiotic dosage but still involve additional formulation costs and monitoring. Plant extracts are generally low–medium cost, described as eco-friendly and practical but requiring optimization for dosing and sourcing. Probiotics are also low–medium cost: they are widely regarded as cost-effective and scalable, but continuous supplementation and quality assurance are needed to maintain efficacy.

Antibiotics remain highly effective in controlling *Vibrio*-related infections but are associated with medium–high costs, antimicrobial resistance, and residue concerns in aquaculture products [58,60,61]. In contrast, plant extracts such as guava leaf extract represent cost-effective and environmentally sustainable alternatives, offering immunostimulatory benefits alongside moderate protection against *Vibrio* species [22,44]. Probiotics also demonstrate encouraging results in reducing mortality and improving shrimp health, often at lower costs, though they require continuous supplementation to maintain their benefits [51,60,62]. Likewise, combined therapies, such as enrofloxacin with San-Huang-San, show strong potential by significantly reducing mortality compared to single agents and allowing for lower antibiotic doses, but still carry considerations related to antimicrobial resistance, herbal standardization, and formulation costs [59]. Taken together, the comparison underscores that while antibiotics remain the dominant tool, plant extracts, probiotics, and integrated antibiotic–plant extract therapies are emerging as promising, cost-effective, and sustainable alternatives that could help reduce reliance on antibiotics in shrimp aquaculture.

#### 2.6.3. Mechanistic Comparison of Antibiotics, Plant-Derived Agents, and Other Alternatives

In aquaculture, antibiotics remain the most widely applied approach against *Vibrio* spp., targeting essential cellular processes. β-lactams and cephalosporins act by inhibiting cell wall synthesis; while tetracyclines, macrolides, and phenicols block protein synthesis, and fluoroquinolones inhibit DNA replication and sulfonamides interfere with folate metabolism [63,64,65]. While effective against planktonic cells, these agents face limitations in biofilm contexts, where reduced penetration and altered metabolic states confer biofilm tolerance, often requiring substantially higher concentrations for eradication than for inhibition [22,66,67]. Moreover, the frequent use of antibiotics in aquaculture has accelerated the emergence of multidrug-resistant *Vibrio* strains, complicating treatment outcomes and raising concerns about the dissemination of resistance genes through the aquatic environment [68]. Residual antibiotics in shrimp tissues also represent a risk for food safety and international trade compliance, which further underscores the need for alternative or complementary strategies [61,69].

By contrast, many phytochemicals and plant-derived extracts exert multi-target mechanisms that make them less likely to induce resistance [66,70,71]. Flavonoids, tannins, and phenolic acids can cause membrane disruption and depolarization, quorum-sensing (QS) inhibition, and down-regulation of virulence factors, thereby limiting bacterial colonization and pathogenicity [70,72]. Other compounds interfere with extracellular polymeric substance (EPS) matrix formation, impairing adhesion and biofilm stability, or modulate oxidative stress pathways via reactive oxygen species (ROS) [73,74]. Recent studies confirm that extracts of guava, moringa, and pomegranate derivatives reduce *Vibrio* virulence gene expression, biofilm biomass, and shrimp mortality at sub-MIC levels [22,75]. Essential oils, such as those from oregano and thyme, have demonstrated strong antibiofilm and bactericidal activity, and several phenolic compounds (e.g., quercetin, punicalagin) have shown synergy with antibiotics [76,77,78]. These features not only broaden their antimicrobial spectrum but also highlight their potential in integrated therapy frameworks, particularly when combined with antibiotics to overcome resistance and improve shrimp survival.

Together, these findings suggest that while antibiotics continue to serve as the backbone of *Vibrio* management, the integration of plant-derived agents provides additional mechanisms that can weaken bacterial defenses and reduce virulence. This complementary nature has motivated increasing attention to combined therapies, where antibiotics and phytochemicals are applied in synergy to enhance treatment efficacy while reducing selective pressures for resistance and lowering the doses required of each agent [59]. This dose-sparing effect is particularly valuable in aquaculture because it reduces both toxicological risks to shrimp and the residues accumulating in shrimp tissues destined for human consumption [23,53,54,59]. Mechanistically, many plant-derived compounds act as adjuvants: essential oils and flavonoids increase membrane permeability, facilitating antibiotic entry; tannins and alkaloids can inhibit efflux pumps, thereby restoring antibiotic susceptibility in resistant *Vibrio* isolates, and phenolic compounds can interfere with quorum sensing, attenuating virulence and making bacteria more vulnerable to antibiotics [44,79,80]. Several studies have confirmed that combinations such as enrofloxacin with herbal mixtures or ciprofloxacin with essential oils produce superior survival outcomes in shrimp challenged with *V. parahaemolyticus* compared to either agent alone [59,81]. Moreover, synergistic combinations not only enhance antimicrobial activity but also limit the selective pressure for resistance emergence, since the bacteria are attacked through multiple targets simultaneously [66,80]. While most evidence still comes from laboratory studies, these findings strongly support the idea that antibiotic–plant extract combinations could form the basis of sustainable integrated management frameworks in shrimp aquaculture, provided that more standardized in vivo trials and cost-effectiveness analyses are performed.

In recent years, probiotics, prebiotics, postbiotics, and synbiotics have emerged as promising alternatives to antibiotics and phytochemicals in shrimp aquaculture. Their protective mechanisms are multifactorial: they compete with pathogenic *Vibrio* for adhesion sites and nutrients, produce antimicrobial metabolites such as bacteriocins, organic acids, and hydrogen peroxide, and stimulate host immunity by activating innate defense mechanisms (e.g., phenoloxidase activity and antimicrobial peptide production). Probiotics can also promote gut microbiota resilience, maintaining a balanced community that indirectly limits *Vibrio* colonization and biofilm formation [82,83,84]. Prebiotics, by providing substrates for beneficial bacteria, further enhance this effect, while postbiotics (non-viable bacterial products) supply bioactive metabolites without the risks of live inoculation. Synbiotics, combining probiotics and prebiotics, are being increasingly studied for their additive benefits [56,85,86]. Recent in vivo studies have shown that *Lactobacillus* spp., *Bacillus* spp., and *Bifidobacterium* spp. supplementation improves shrimp survival against *V. parahaemolyticus* and *V. alginolyticus* challenges, while also enhancing growth performance and antioxidant capacity [60,83,87]. Other promising candidates include yeast-derived products and marine actinobacteria, which have demonstrated immunostimulatory and antivirulence properties [88,89]. Importantly, some probiotics not only reduce mortality but also decrease biofilm formation and virulence factor expression, complementing the antibiofilm role of plant extracts. Nevertheless, as indicated in Table 7, the main limitation of these strategies is the need for continuous supplementation to maintain their beneficial effects, which may raise operational costs and affect scalability [60]. In this context, attention has recently shifted to antimicrobial peptides (AMPs) as another biologically derived tool with the potential to complement existing probiotic and phytochemical strategies. AMPs, particularly those derived from marine bacteria and aquaculture-associated microbiomes, represent a novel mechanism-distinct option. They act primarily by disrupting bacterial membranes, destabilizing biofilm structure, and in some cases interfering with quorum sensing [88,90,91]. Importantly, AMPs can be integrated with existing strategies: they have shown synergy with antibiotics, plant extracts, and even probiotics, offering dose-sparing and enhanced antibiofilm activity [90,92]. While challenges remain in production cost and stability, recent studies demonstrate their efficacy against multidrug-resistant *V. parahaemolyticus*, *V. alginolyticus* and other *Vibrio* species in experimental shrimp models [93,94,95]. This highlights AMPs as promising candidates for inclusion in combined therapy frameworks, complementing antibiotics, phytochemicals, and probiotics as part of sustainable *Vibrio* management.

## 3. Discussion

Shrimp farming is one of the fastest-growing industries and represents significant economic importance worldwide. In Ecuador, it is the main aquaculture activity, as this is the main country producing shrimp worldwide. This industry principally involves the production of *P. vannamei* or whiteleg shrimp, which is cultured in 21 countries and is the primary species reported in the Americas [96]. However, shrimps are highly susceptible to diseases, and in recent years, various problems have significantly affected shrimp production and health, with vibriosis being one of the major issues [97]. *Vibrio*-related infections are caused by multiple species of the facultatively anaerobic, Gram-negative genus *Vibrio*. *V. parahaemolyticus*, *V. alginolyticus*, and *V. harveyi* are among the *Vibrio* species most isolated and identified in diseased shrimp, as reported in the literature [56]. Therefore, this systematic review was conducted to analyze the strategies currently used to treat different *Vibrio*-related infections, identify the most prevalent species, and highlight important points for the culture of *P. vannamei* shrimp.

Through the analysis of our data, various strategies were identified to mitigate infections related to *V. parahaemolyticus* and *V. alginolyticus* in addition to other species of *Vibrio,* especially for vibriosis in Pacific white shrimp (*P. vannamei*) were identified in different geographic regions. The analysis indicated a high prevalence of types of *Vibrio* in shrimp, with the highest prevalence reported in North America (100%), followed by Asia (84.89%) and South America (74.14%). The most common *Vibrio* species were *V. parahaemolyticus* and *V. alginolyticus*, although other species were identified. In comparison with other studies, it is observed that the prevalence of *Vibrio* and their species varies in Asia, ranging from 10.47% to 47.6% according to certain reports, but other studies stated almost 100%, differing also to the American continent studies, which report a prevalence of 60% in most articles [98,99]. In the majority of the worldwide studies, the predominant species causing vibriosis in shrimp are *V. parahaemolyticus* and *V. alginolyticus*, followed by other *Vibrio* species such as *V. cholerae* and *V. vulnificus* [100,101,102,103]. However, the variation in prevalence among continents, with a higher incidence in Asia compared to America, could be attributed to environmental factors, farming practices, and disease control strategies implemented in each region, leading to varying levels of antibiotic resistance [104].

This review highlights that while multiple strategies have been investigated to control *Vibrio*-related infections in *P. vannamei*, their effectiveness varies substantially depending on whether pathogens are in planktonic or biofilm states, the compound applied, and the farming context. Antibiotics remain the backbone of disease control because of their broad availability, established protocols, and rapid action. However, across the studies reviewed, their consistent limitations were reduced efficacy against biofilms, the rapid emergence of resistant strains, and the persistence of resistance genes in aquatic environments [105,106,107,108,109,110]. This not only undermines treatment success but also creates a direct link between aquaculture and global antimicrobial resistance challenges, reinforcing that antibiotic-only approaches are unsustainable in the long term.

In contrast, plant-derived agents and phytochemicals provide multi-target activities that include membrane disruption, quorum-sensing inhibition, and biofilm destabilization [22,55,66,70,71,72,73,74,75,111]. Their ecological origin makes them attractive alternatives, yet reproducibility across studies remains inconsistent due to variation in plant sources, extraction methods, and compound concentrations. Without standardized protocols for extraction, dosing, and testing, the translation of promising laboratory results into reliable field applications will remain limited. Still, the observation that some extracts not only reduce *Vibrio* virulence but also enhance shrimp survival suggests that these agents could serve as effective adjuvants in integrated strategies rather than as stand-alone treatments [22,55,77].

Combined therapies, particularly the use of antibiotics together with plant extracts or isolated phytochemicals, emerge as one of the most promising strategies identified in this review [59,76,77,78,80,81,82]. These combinations can enhance antimicrobial efficacy, reduce the required antibiotic dose, and slow the development of resistance by targeting bacteria through complementary mechanisms. Beyond these synergies, an important research opportunity lies in expanding the scope of combination strategies to include not only antibiotics but also emerging alternatives such as postbiotics and antimicrobial peptides (AMPs) [56,85,86,87,88,89,90,91,92,93,94,95]. Plant compounds with antivirulence and antibiofilm properties could potentiate the effects of these newer biological tools, providing a multi-layered approach that integrates conventional treatments with ecological and sustainable innovations. The challenge ahead is to validate these combinations under standardized in vivo conditions in shrimp, ensuring reproducibility, scalability, and cost-effectiveness for practical aquaculture application [51,55,77,112].

Taken together, these patterns highlight that the sustainable control of *Vibrio*-related infections in shrimp aquaculture will likely depend less on single agents and more on rationally designed combinations that balance efficacy, safety, sustainability, and cost. Importantly, this review also revealed a key limitation: most of the available evidence comes from Asia and South America, with very limited data from Africa, Europe, and North America [98,99,100,101,102,103,104]. This geographic imbalance constrains the generalizability of findings and underscores the need for research efforts in underrepresented regions to capture a broader diversity of farming practices and environmental conditions.

Future work should focus on (i) establishing standardized procedures for in vivo testing of antibiotics, plant extracts, probiotics, postbiotics, and AMPs in shrimp models, (ii) integrating cost–benefit analyses into efficacy studies to ensure practical scalability, (iii) exploring regional differences in *Vibrio* prevalence and resistance dynamics, and (iv) prioritizing combination therapy designs that exploit synergies while minimizing resistance development. Addressing these gaps will be essential not only for improving shrimp health and productivity but also for ensuring that aquaculture aligns with One Health goals of reducing antimicrobial resistance and promoting sustainable food systems.

## 4. Materials and Methods

### 4.1. Literature Search

#### 4.1.1. Literature Search *Vibrio* Treatment with Antibiotics

This study was conducted following Preferred Reporting Items for Systematic Reviews and systematic review (PRISMA) guidelines [113]. The Scopus and PubMed databases were searched for journal articles, in English, using combinations of Boolean terms and Medical Subject Headings (MESH). Specifically, the searched terms and combinations were as follows: “*Vibrio parahaemolyticus*”, “*Vibrio alginolyticus*”, “*Litopenaeus vannamei*”, “*Penaeus vannamei*”, and “treatment”. Articles that reported results on antimicrobial tests of different antibiotics against *Vibrio* pathogens, specifically the two species *V. parahaemolyticus* and *V. alginolyticus* of interest in the agricultural and shrimp industry, were positively selected. The references to these articles were also checked to find additional records. All references were compiled into a Zotero Library database (www.zotero.org) and then managed using Excel software.

#### 4.1.2. Literature Search *Vibrio* Treatment with Plant Extracts

The PubMed database was searched for journal articles, in English, using combinations of Boolean terms and MESH terms. The searched terms and combinations were as follows: “*Vibrio*”, “*V. parahaemolyticus*”, “*V. alginolyticus*”, and “extract”. Articles reporting results on antimicrobial tests using different plant extracts against the two *Vibrio* species of interest were considered. The references to these articles were also checked to find additional records. All references were compiled into a Zotero Library database (www.zotero.org) and then managed using Excel software.

### 4.2. Screening Process

Duplicates were initially identified and removed in Zotero after entering all the recognized studies into a self-created Excel database (see File S1). All articles were assessed by three reviewers (AB-N, NRJ-M, and ACC-P) by screening titles, abstracts, topics, and finally full-length texts. At each level, the reviewers independently screened the articles and then merged their conclusions. An additional examination of the selected articles was conducted by two other reviewers (ET and AM) to ensure the homogeneity of the eligibility criteria applied by the previous reviewers in the initial dataset. Discrepancies were resolved by discussion before finalizing the records for the evaluation of eligibility criteria (see next subsection). In case of disagreements, a third assessor (AM or ET) was assigned to make a final decision.

### 4.3. Eligibility Criteria, Data Extraction, and Quality Assessment

The main inclusion criteria were the results of antimicrobial assays with different types of antibiotics and plant extracts. Data on the most used antibiotics and different types of plant extracts, *Vibrio* species, and methodologies were extracted if available. Duplicate reports from different databases were excluded from the final dataset for the systematic review. Finally, articles without full-length text available and studies with missing or incomplete data were also omitted. The extracted information included the first authors’ names, year of publication, location, *Vibrio* species, antibiotic names, methodologies, plants, types of extract, and quantifiable parameters (effectiveness, survival rate, and number of replicates). The last three parameters were the most critical methodological criteria in the initial screening. Additionally, studies involving assays with methodologies other than agar diffusion method were identified for further evaluation. The initial three authors (AB-N, NRJ-M, and ACC-P) extracted all data, and further confirmation and evaluation were conducted by the lead authors (ET and AM). The final document with the collected data is available upon request from the authors.

### 4.4. Plant-Compound Relationship Identification and Molecular Analysis

For the identification of chemical compounds present in the plants obtained from the literature review, we explored the following publicly available databases (accessed on 1 February 2024): LOTUS [114], COCONUT [115], Korean Traditional Knowledge Portal (KTKP) (https://www.koreantk.com/ktkp2014/), IMPPAT: Indian Medicinal Plants [116], and ETM-DB (http://biosoft.kaist.ac.kr/etm).

The compounds extracted from these databases are presented in several formats. These formats include SMILES representation, SDF data information, or only the molecule names. The RDKit (RDKit: Open-source cheminformatics, https://www.rdkit.org, accessed on 1 March 2024) and PubChemPy (https://github.com/mcs07/PubChemPy, accessed on 1 March 2024) python packages were used to homogenize representation to SMILES strings and to remove duplicated molecules. Additionally, all molecules were classified using NP classifier version 1.5 (https://npclassifier.ucsd.edu/, accessed on 1 March 2024) [117], according to their chemical classes and subclasses.

## 5. Conclusions

This systematic review provides comprehensive insights into strategies for managing *V. parahaemolyticus* and *V. alginolyticus* infections in whiteleg shrimp. While antibiotics remain the most widely used intervention, the analysis shows that only certain families, particularly phenicols, cephalosporins, and carbapenems, retain consistent activity against these pathogens. Their use, however, must be restricted and guided by laboratory diagnosis to avoid further resistance development.

Among natural alternatives, plant extracts rich in flavonoids, tannins, and phenolic acids (such as guava (*Psidium guajava*), moringa (*Moringa oleifera*), and pomegranate derivatives) emerged as the most promising candidates, with evidence of reducing virulence gene expression, biofilm biomass, and shrimp mortality. These agents, if standardized for dosage and extraction protocols, could be implemented as sustainable feed additives in shrimp farming.

Combined therapies represent the most immediately applicable innovation. Integrating antibiotics with phytochemicals allows dose reduction, improved survival outcomes, and lower selective pressure for resistance. Practical examples include the combination of enrofloxacin with herbal formulations or ciprofloxacin with essential oils, which have shown superior protection in experimental infections. Such combinations could be adapted into farm protocols with proper cost–benefit evaluation and safety validation.

Looking ahead, stakeholders should prioritize (i) adopting combined strategies that integrate antibiotics with validated plant-based agents, (ii) supporting the development of standardized protocols for extract preparation and in vivo testing, and (iii) monitoring regional prevalence and resistance trends to guide antibiotic family selection. This tiered approach offers the most feasible path toward sustainable *Vibrio* management in shrimp aquaculture, reducing reliance on antibiotics while ensuring shrimp health, farm productivity, and food safety.

## Figures and Tables

**Table 3:** Analysis of different types of *Vibrio* observed in various geographical regions.

Region	Country	Number of Studies	*Vibrio* Presence Rate	Prevalence of *Vibrio* (%)
Asia	Thailand Vietnam Taiwan Indonesia Korea Malaysia India Singapore China	21	6038/7112	84.89
South America	Brazil Ecuador	4	353/474	74.47
North America	Mexico	1	180/180	100

## Data Availability

The data that supports the findings of this study are available in the main text of the manuscript and Supplementary Material. Further information can also be given on request from the corresponding authors.

## Supplementary Materials

The following supporting information can be downloaded at: https://www.mdpi.com/article/10.3390/molecules30173620/s1, File S1. *Seleccionador*, which constitutes the Excel database containing the data collected in this study.

## Abbreviations

The following abbreviations are used in this manuscript:

AHPND	Acute hepatopancreatic necrosis disease
WFD	White feces disease
SGS	Seagull syndrome
HGT	Horizontal gene transfer
PRISMA	Preferred Reporting Items for Systematic Reviews and systematic review
MESH	Boolean terms and Medical Subject Heading
KTKP	Korean Traditional knowledge portal
IMPPAT	Indian Medicinal Plants
